# A Rare Presentation of a Bi-Maxillary Brown Tumour due to Secondary Hyperparathyroidism

**DOI:** 10.1155/2023/6180006

**Published:** 2023-03-27

**Authors:** Cheboh Cho-Fon, Zilefac Brian Ngokwe, Obolo Nwaga Ines

**Affiliations:** Faculty of Medicine and Biomedical Sciences, University of Yaounde 1, Yaounde, Cameroon

## Abstract

Brown tumours are localized bone lesions, seen in patients with high parathyroid hormone levels. This can be due to primary hyperparathyroidism, which occurs more often in neoplasms of the parathyroid gland or due to secondary hyperparathyroidism more often seen as a result of renal impairment. Facial involvement is rare, with most reports focusing on long and axial bones. However, the mandibular bone is often the only bone affected. Here, we report a rare case of a bi-maxillary attainment of brown tumour in a patient with secondary hyperparathyroidism due to chronic kidney disease.

## 1. Introduction

Mineral bone diseases are frequent in patients with renal impairment [1] with a prevalence of about 58% [2]. These bone lesions can be a result of calcium–phosphorus imbalance due to kidney malfunction, resulting in secondary hyperparathyroidism (SHPT). Most reports on mineral bone disease have focused on long and axial bones. The true burden of mandibular involvement in SHPT is unknown [3]. The bony lesions are due essentially to increased circulating levels of parathyroid hormone, which have osteoclastic activities primarily in cortical bones. This may explain why the mandible, a cortical bone, is the most commonly affected site in the maxillofacial area, whereas maxillary involvement is less common [4]. Late bony manifestations of the disease include generalized osteoporosis, multiple focal areas of demineralization of the skull, and osteitis fibrosa cystica (brown tumour) [4]. The manifestations of SHPT in the mandible are bone demineralization, decreased trabeculation, “ground glass” appearance, periodontal defects, metastatic soft-tissue calcifications, loss of lamina dura, pulpal narrowing and calcification, and brown tumours [3]. Here, we present a case of bi-maxillary attainment of brown tumour in a patient with SHPT and end stage renal disease on maintenance hemodialysis.

## 2. Case Presentation

We present the case of a 35-year-old male with a body mass index of 20.9, non-smoker, hypertensive (190/98 mmHg; Grade 3 WHO) on calcium channel blockers (amlodipine), diagnosed with Chronic kidney disease in 2007, and has been on dialysis (twice a week, 4 hours each) since then. During the dialysis sessions, he received vitamin D supplements. His serum calcium level was 8.7 mg/dl (normal range, 8.8–11 mg/dl), phosphate was 4.5 mg/dl (normal range, 2.5–5.0 mg/dl), and Parathyroid hormone level was 2356 pg/dl (normal range, 15–65 pg/dl).

**Figure 1:**
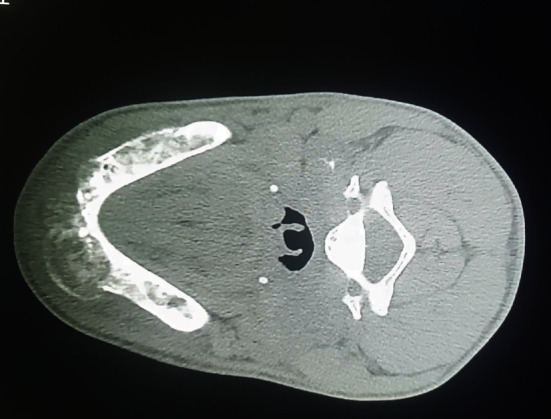
Mandibular bone attainment (self-taken).

**Figure 2:**
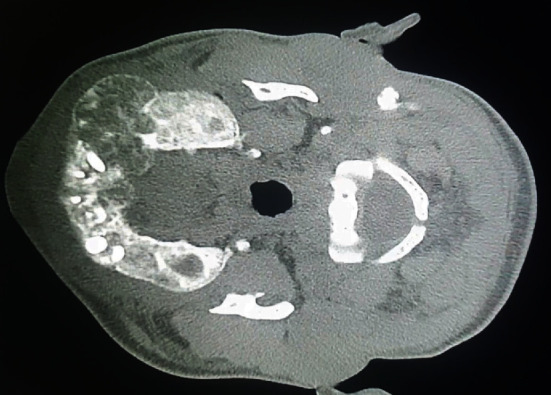
Maxillary bone attainment (self-taken).

The patient presented a severe bilateral facial swelling, which started insidiously about 3 years ago. He complained of pain during mastication and speech difficulties. An initial diagnosis of brown tumour was posed with a differential diagnosis of ameloblastoma and osteofibroma pending imaging and biopsy. He received vitamin D and phosphate binders as treatment. A CT scan performed showed a bi-maxillary attainment of the jawbones, as shown in Figures 1 and 2.

The diagnosis of brown tumour was confirmed by a histological analysis of a biopsy of the tumour mass. Current practice is a partial removal of the parathyroid gland in the case of SHPT to permit the continual secretion of the parathyroid hormone to control calcium level [5]. A partial (subtotal) parathyroidectomy was carried out 3 years prior with 2 of the 4 parathyroid glands left in place to enable normal parathyroid hormone levels. Unfortunately, parameters failed to return to normal with the PTH level remaining high at 1633 pg/dl, and the mass size did not reduce. He was continued on vitamin D supplementation and phosphate binders in view of further surgery to correct his hyperparathyroidectomy. A resection of the tumour is envisaged if parameters do not improve.

We, unfortunately, lost the patient in 2021 due to the complications of his renal disease before we could do a total parathyroidectomy of a resection of the tumour.

## 3. Discussion

Brown tumours are of epidemiological interest as the prevalence of chronic kidney disease in Cameroon ranges from 10.0% to 14.2% [6–8], and given chronic renal failure is the main cause of SHPT [4]. Chronic kidney disease is both a cause and consequence of non-communicable diseases (NCDs) [9, 10]. More so, poorly controlled diabetes mellitus, hypertension, infection, herbal and environmental toxins, and self-medication play an essential role in the epidemiology of CKD in our local settings [11–13]. Furthermore, the burden of CKD in low-income and middle-income countries (LMICs) is worsened by limited accessibility to and affordability of renal replacement therapy (RRT) [14]. Chronic kidney disease disproportionately affects LMICs than that in high-income countries [15]. As a result, complications such as SHPT would be more prevalent. With maxillary bone brown tumour involvement, the bone lesion usually regresses after parathyroidectomy. However, in some instances, the bone brown tumours can continue to grow despite parathyroidectomy as was the case with our patient [16]. Radiographically, resorption of the lamina dura around the roots of the teeth and demineralisation of the medullary bones of the jaws causing a characteristic “ground glass” appearance [17] as we also observed. Complications and the impact of brown tumours have been noted. In most cases involving the maxillofacial region, patients present with facial deformations, chewing difficulties, speech difficulties, and in the worst cases, asphyxia can occur [18]. Our case had severe facial deformation and chewing problems but had no breathing problems. Resection is often not done as the tumour spontaneously regress once the parathyroid hormone levels become normal [18]. It is envisioned only in case the patient wishes for rapid recovery and when it compromises vital functions [19]. Our case was not operated as the complications of his renal disease ultimately overwhelmed him. Unfortunately, we lost our patient, and SHPT has been shown to be associated with increased mortality in patients undergoing maintenance dialysis treatment [20, 21].

## 4. Conclusion

The case we had was particular because the parathyroid hormone level remained high despite a partial parathyroidectomy. This most probably explains why the mass failed to regress up to 2 years post-surgery. Monitoring the parathyroid hormone levels of patients with renal impairment is essential in preventing these brown tumours and must thus be done often.

## Data Availability

All the data in our manuscript can be found in online journals as stated in the references.
